# “With every passing day I feel like a candle, melting little by little.” experiences of long-term displacement amongst Syrian refugees in Shatila, Lebanon

**DOI:** 10.1186/s13031-019-0228-7

**Published:** 2019-10-10

**Authors:** Hanadi Syam, Emilie Venables, Bernard Sousse, Nathalie Severy, Luz Saavedra, Francois Kazour

**Affiliations:** 1 Médecins Sans Frontières -Belgium, Mission in Lebanon, Ayoub Bldg Bloc A, 6th Floor, Furn Al-Hayek Str Achrafieh, Beirut, Lebanon; 2 Médecins Sans Frontières, Luxembourg Operational Research Unit (LuxOR), Operational Centre Brussels, Luxembourg City, Luxembourg; 3 Division of Social and Behavioral Sciences, School of Public Health and Family Medicine, University of Cape Town, Cape Town, South Africa; 4 Médecins Sans Frontières, Medical Department, Operational Center Brussels, Brussels, Belgium; 5 Department of Psychiatry, Saint-Joseph University, Beirut, Lebanon; 6 Psychiatric Hospital of the Cross, JalEddib, Lebanon

**Keywords:** Long-term displacement, Syrian refugees, Lebanon, Shatila camp, Family dynamics, Gender roles, Mental health, Violence

## Abstract

**Background:**

Long term displacement and exposure to challenging living conditions can influence family dynamics; gender roles; violence at home and in the community and mental well-being. This qualitative study explores these issues as perceived by Syrian refugees who have been living in Shatila, a Palestinian camp in South Beirut, Lebanon, for at least 2 years.

**Methods:**

Twenty eight in-depth interviews with men and women were conducted between February and June 2018. Women were recipients of mental health services, and men were recruited from the local community. Interviews were conducted in Arabic, translated, transcribed, coded and analysed using thematic content analysis.

**Results:**

Our results show patterns of harsh living conditions similar to those described earlier in the course of the Syrian refugee crisis. Lack of infrastructure, overcrowding, cramped rooms and violence were all reported. Participants also described a lack of social support, discrimination and harassment within the host community, as well as limited social support networks within their own Syrian refugee community. Family dynamics were affected by the increased responsibilities on men, women and children; with additional economic and employment demands on men, women assuming the roles of ‘mother and father’ and children having to work and contribute to the household. Participants discussed several types of violence, including parental violence against children and violence in the community. Violence against women was also reported. Reported mental health issues included depression, anxiety, sadness, frustration, hopelessness, self-neglect and a loss of sense of self and self-worth. Some participants expressed a wish to die.

**Conclusions:**

This study describes experiences of changing gender roles, family dynamics, violence and mental health after long-term displacement in in Shatila camp, South Beirut as perceived by Syrian refugees. A lack of safety and security coupled with economic hardship rendered refugees even more susceptible to exploitation and harassment. Parental violence was the most commonly reported type of domestic violence.

## Background

Since the onset of the Syrian war in 2011 and the ensuing refugee crisis, a large number of Syrians have been displaced and have since found refuge in neighbouring countries. As of September 2016, the number of displaced Syrians reached approximately five millions; globally being one of the main three countries from which more than half of refugees come (United Nations High Commissioner for Refugees [25], p.3). Lebanon had an estimated population of six million inhabitants as of the year 2018 with almost one million registered refugees, in addition to an undetermined number of unregistered refugees [26].

Syrian refugees have resettled in a variety of locations across Lebanon including existing Palestinian refugee camps where living conditions are harsh and inadequate. There are also an estimated 450,000 Palestinian refugees within Lebanon, 53% of which live in 12 camps across the country [28]. Shatila refugee camp, where this study took place, is one camp with immense overcrowding and extremely limited access to resources including housing, employment, electricity and water [12, 20]. In 2015, the ‘open-door policy’ for refugees from Syria which included not needing a visa for entry and free of charge residency renewals came to an end after lengthy and costly residency renewal regulations were issued by the Lebanese government (Human Rights [14]). As a result, many Syrian refugees lost their legal status, which in turn restricts their movement, employment and access to basic services, including health services (Human Rights [14]).

The mental health status of many Syrian refugees who have been displaced in Lebanon for many years is of concern, and research in several settings have shown that long term displacement and exposure to challenging living conditions can have negative consequences on mental health [4, 29]. Women in particular have been shown to be under enormous stress out of concern for the well-being of their children and the pressures of poverty, unemployment and debt [22]. Violence towards Syrian refugees has also been reported in other contexts as a consequence of long term displacement [18]. Long-term displacement can also have consequences on family dynamics, such as shifts in gender roles and changes in employment patterns between men and women which have been noted in other studies from Lebanon [8, 22].

Access to health-care is challenging and limited for Syrian refugees, with a particularly acute lack of specialised services in camps, including Shatila [12]. Mental health services are especially lacking [19]. In 2013, the international medical humanitarian organisation *Médecins Sans Frontières* (MSF) responded to the needs of Syrian refugees into Palestinian camps around Beirut by opening a Primary Health Centre (PHC) and a Women’s Health Centre (WHC) in Shatila, which included the provision of mental health services.

Since 2015, MSF staff working in the mental health clinics in Shatila observed that patients expressed suffering that they linked more to the effects of long-term displacement in Shatila than the acute effects of war. Anecdotal data from MSF psychologists also indicated increased cases of domestic violence compared to earlier periods of the Syrian refugee crisis; prompting further exploration. We wanted to understand more about the Syrian refugees who had been living in Shatila for a prolonged period through exploring their living experiences, perceived changes in family dynamics and gender roles, experiences of violence, and their mental health concerns. Whilst qualitative research has been conducted with similar refugee populations within Lebanon [22], this question remains as yet unanswered within this urban camp context.

Our research study explores the Syrian refugees’ living experiences in Shatila camp following long-term displacement, defined for the purposes of the study as someone spending at least 2 years in Shatila, including perceived changes in family dynamics, gender roles and experiences of violence within the household as well as experiences of mental health.

## Methods

### Study design

This is a qualitative research study, involving in-depth interviews (IDIs) with adult male and female Syrian refugees living in Shatila refugee camp in Southern Beirut, Lebanon.

### Shatila refugee camp

Shatila refugee camp in South Beirut covers an area of 1km^2^. It was established in 1949 by the International Committee of the Red Cross (ICRC) to accommodate hundreds of refugees coming from various regions in northern Palestine [28]. Today, the unofficial number of inhabitants in Shatila is around 40,000, including an estimated 18,000 refugees from Syria who have arrived since the Syrian crisis began in 2011 [21]. Security inside the camp is loosely controlled by a local security committee consisting of representatives from the different Palestinian political factions in the camp [11]. This particular study site was chosen as MSF provides mental health services for Syrian refugees in this setting, and wanted to learn more about the population through conducting the study in Shatila.

### Study population

All study participants were Syrian refugees aged over 18 who had resided in Shatila refugee camp for a minimum of 2 years (as verified by the research team when explaining the study to potential interviewees) and who defined themselves as being married. All female participants were beneficiaries of MSF’s mental health services, excluding those with severe or acute psychological conditions. Male participants were not beneficiaries of MSF’s services, as there were very few men attending the clinic for mental health support. No husband and wife couples were included in the study.

### Recruitment

Purposive sampling was used to recruit female participants who met the eligibility criteria of the study. Two members of the psycho-social team who were not involved in the research study consulted patient registers to check the eligibility of female mental health patients for the study.

Potential female participants identified through the register were briefed by the psycho-social team on the study and its objectives making clear that the study was a separate activity, distinct from the mental health services they were currently receiving or had previously received, and that they were not obliged to participate. Care was taken to make sure that study recruitment did not interfere with therapeutic care, and that the two teams were kept distinct. If women were interested in participating, they were invited for an interview on the same day, after the end of their mental health session, or were given a different date and time if they preferred. No member of the research team was involved in providing mental health care to the research participants. Male and female participants were offered a follow-up psychological support after the interview to discuss any potential distress that may have occurred during interviews.

Convenience sampling was used for male participants who were also required to be over 18, married and have been living in Shatila for at least 2 years. They were approached by the health promotion and social work team (of which the principal investigator is part) in the waiting area outside the WHC, where they waited for their wives to finish their clinical consultations and were invited to take part in an IDI. As with the female participants, the study objectives were explained to the male participants, along with the voluntarily nature of their participation. Some of the men approached refused to take part in the study, but the reasons behind this refusal were not probed so as not to inconvenience them further. The IDIs then took place at a date and time identified by the participant.

### In-depth interview procedures

In-depth interviews were conducted by the Principal Investigator (PI) in a room in the MSF office in Shatila, as this was a place which participants were familiar with and one of the few spaces which can provide privacy. All interviews were audio-recorded with the consent of the participant. No participant refused to be audio-recorded. All participants were interviewed once and no repeat interviews were conducted.

Interviews were conducted using an in-depth interview guide formulated with open-ended questions. Men and women were asked similar questions; with additional questions for women about their reasons for seeking mental health services (see Additional files 1 and 2). The interview guides were pre-tested with one male and one female interviewee before conducting the interviews, to ensure that the questions were easy to understand and yielded answers relevant to the study objectives. The interviews conducted during the pre-testing were not included in the final analysis. Handwritten notes were also taken by the PI during the interviews. Interviews were conducted in Arabic (the first language of the PI and all of the participants), and the average duration of interviews was 38 min (minimum: 20 min, maximum: 83 min).

Interviews were carried out with a total of 14 men and 14 women over a four-month period, between February and June 2018. Interviews were stopped when saturation was reached, meaning when interviews did not reveal any new information from the research participants (O [16]). In this case, the PI was hearing very similar descriptions of life in Shatila, insecurity and mental health issues but conducted further interviews to probe for examples of violence until saturation was reached.

A co-investigator was present during the first four interviews in order to give input into the data collection and suggest any areas of improvement or amendment to the questions or style of interviewing. The PI and the co-investigator supporting the initial interviews were both female. In one interview, a male member of the MSF health promotion team who assisted with recruitment for the study was present at the request of the participant.

No financial reimbursements were provided, but participants were offered refreshments during the interview.

Audio-recordings were translated from Arabic into English and transcribed by external, professional transcribers. Transcription and translation were done in a ‘one step’ process in which the Arabic recordings were transcribed directly into English.

Transcripts were read, annotated, reviewed and manually coded by two co-investigators of the study. Such review also allowed for the improvement of subsequent interviews, as areas for further probing or questions were pointed out.

Any discrepancies detected during the review by the two co-investigators were resolved through ongoing discussion amongst the research team, and referring back to the original Arabic audio-recordings for verification where necessary. Preliminary, anonymised findings were also shared and discussed with other members of the MSF team working in Shatila and the other co-investigators for verification.

### Data analysis

Thematic content analysis was used, in which data in the transcripts were organised, coded, categorised and interpreted. Data was analysed in an inductive way, in which codes were generated from the data that was collected during the interviews, and themes were then extracted from these codes. Codes were attached to statements from the transcripts in order to structure the data [15].

### Ethical issues

This protocol was approved by the MSF Ethics Review Board (reference 1705) and the Ethics Committee of University of Saint Joseph in Beirut, Lebanon (USJ-2017-67). Written informed consent was taken in Arabic from all participants before data collection began. Participants were given detailed information during the informed consent process to ensure that they did not think that participation in the research study would impact their access to MSF-provided health-care, or could lead to financial benefits. All identifying data was removed from transcripts.

## Results

Our results are divided into four main themes, all described from the perspective of male and female Syrian refugees: 1) experiences of living in Shatila refugee camp, 2) perceived changes in family and gender dynamics since displacement from Syria, 3) experiences of violence and 4) experiences of mental health. A total of 28 participants were involved in the study: 14 were male and 14 were female. Participants had a mean of 3.7 children. The number of years since displacement ranged from two to 7 years, with a mean of 4.2 years living in Shatila camp (see Appendix: Table 1 for detailed profile of study participants).

### Experiences of living in Shatila

#### “*We used to live freely*”: life in Syria before displacement

Male and female Syrians described their life in Syria before the crisis. Many were from rural areas and talked about the open spaces, green landscapes and fresh air, and how they saw Syria as a safe place before the conflict. Women in particular described how they frequently visited their neighbours and spent time in the fresh air outside:

In Syria we have fresh air and sun and we can sit in front of our houses. Here we don’t have any space. The door is always closed. Sometimes the children want to play outside the room but I don’t allow them to…there is air but sunlight does not enter. (Female, aged 26)

Participants, including the 42 year old male cited below, described how they could access everything they needed in Syria:

In Syria if I broke both legs I wouldn’t care because we could have anything. Here you always have to fight to live.

Whilst people fled because of the conflict, many including this 35 year old male participant expressed a desire to return: “*living here is worse than shelling…hunger is way worse than the shelling*.”

#### *“We eat from the trash”*: living conditions in Shatila

All participants talked about the miserable and inhumane living conditions they experienced in Shatila. They described living in cold, dark, cramped rooms without running water or electricity and one 37 year old female interviewee described Shatila as “*ghabet w7oosh mesh bashar*”[so savage it cannot be for humans].

Many Syrian refugees shared one room with several family members, and one participant’s son slept in the kitchen as they had limited space. There were several reports of increased health issues such as respiratory problems as a result of damp houses and lack of sunlight. In turn, their financial situation meant they could not afford to access health-care outside of MSF’s services.

One recurrent theme during interviews was the daily struggle for food, with multiple participants talking about how they eat food from the garbage bins in the camp:

My husband brings us food from the big vegetable market. I think he’s getting it from the trash there… I swear we eat from the trash, we eat rotten tomatoes. (female, aged 25).

Cars come and sell vegetables in the suburbs. Whatever extras they throw away I bring to my family. (male, aged 59).

Men in particular described the challenge of finding work, and the difficult working conditions they faced if they were able to secure employment. Interviewees felt that the Syrian conflict and increase of refugees arriving created additional competition for employment, which in turn reduced earnings and job security.

The insecure and unsafe environment in Shatila was a very prominent theme during interviews with men and women. Participants were scared to go outside in the streets and many kept their children locked inside their rooms. One 59 year old male participant compared his current living conditions to the Syrian conflict:

I ran away from a war that is clear and direct, to come here to a war that could erupt at any second. It feels like being around a time-bomb… You don’t know when it will explode.

A 37 year old woman described how her daughter suffered and developed post traumatic symptoms after being kidnapped within the camp:

My 6 year old daughter was once kidnapped from the school. They took her to a basement, took off her clothes, put cotton in her mouth and showed her porn movies… She’s not going to get back to the way she was, she gets stressed whenever she sees little boys. She also hated her brothers for a long period of time and she was afraid of them. She gets a fever when she remembers these things…she throws her toys, runs to her bed and covers her face and tells me she wants to sleep.

All participants talked about their economic challenges and the exorbitant cost of rent in the camp. They described feeling trapped inside their rented rooms and confined in Shatila. Many referred to the camp as a prison with one 61 year old man describing it as a place where he ‘*cannot get a breath of fresh air’* and another interviewee saying they felt that they were ‘*in a grave’*. Some women made infrequent visits to a local park with their children, but most people did not leave Shatila. Challenges in renewing their residency permits meant that they were fearful of being stopped if they went outside.

#### “*They made us hate our lives*”: community and support networks in Shatila

Men and women described the lack of community and support networks in Shatila compared to their networks in Syria:

*[N]o one is there for the other… If someone gives you something, they always want something in return.* (female, aged 31).

Women in particular compared their lack of social activities and friendships in Shatila to the strong bonds they had in Syria. They had little contact with their neighbours in the camp and were often in conflict with those living around them, including their landlords. Participants reported tensions with Palestinians living in the camp; their Lebanese landlords and employers and other Syrians.

The cramped living conditions and insecure context meant that interviewees had reduced contact with their neighbours, and limited support and friendship networks:

Here it’s a prison. We’ve been here for 2 years and nobody came and asked how we were doing. My wife once said ‘send us back to Syria, we are suffocating here…look at the children, they are not growing up!’ (male, aged 35).

We used to look at the aircraft above us and hide; here we hide from people. (male, aged 36).

#### “Whatever we do, they talk trash about us”: harassment in Shatila

Interviewees reported several types of harassment in Shatila, including racism and discrimination from non-Syrians, bullying, blackmail, verbal and physical abuse and in the case of women, sexual harassment in the street. They also described the bullying and harassment that their children received from other children and adults in the camp.

There were several examples of men being blackmailed:

How is it possible that if I put a box and sell some cigarettes in the street, he demands $50 a week from me? Just because I was sitting opposite his shop! (male, aged 59).

Two women talked about how their husbands were the victims of physical and psychological violence and humiliation:

My husband was beaten twice but no one dared to help him. Syrians are mistreated here. We have to shut up, we’ve got no other option. (female, aged 40).

Three years ago my husband was on his way back from work. Some bad guys released a dog to bite him and took his clothes off. They took his wallet and broke his ID. (female, age 38).

### Family and gender dynamics

#### “She’s taking the roles of mother and father.” changes in traditional gender roles

Men and women felt that there were changes in traditional gender roles and responsibilities, with men and women feeling that women were taking on roles previously associated with men. Notably those changes related to parenting and responsibilities towards children. The composition of the family also changed for many Syrians, who were sharing very cramped rooms with multiple family members, including their children.

A 37 year old female interviewee described how her life had changed after displacement:

Here, it’s not like in Syria…women have to cook, go to market, take kids to doctors… There, I was only involved when my kids went to hospital, I was not allowed to do more.

More than one participant referred to men and women playing the role of both genders:

Here I am a man and a woman. If the kids are sick I have to take them for medical care, when they call from school I’ve got to go. I’ve got to get the food, I’ve got to cook and clean… I’m responsible for everything. I feel for my husband because he works all day and comes back at night, but he still can’t get me everything that I need. (female, aged 44).

[S]he’s taking the roles of the mother and the father. (male, aged 35).

[M]y wife has to continue going around and attending social activities organized by some NGOs to get some incentives in return… In Syria she did not need to come and go…she used to stay at home as a housewife (male, aged 61).

Men’s financial responsibilities intensified when their families joined them in Shatila and they often found themselves working long hours and only seeing their families in the evenings, as the following examples show:

Here I’m working more than I can bear to. I’m spending more time at work than I am with my family. (male, aged 25).

The woman is playing the role [of a parent] all day while the man is playing it only at night. (male, aged 21).

Many male interviewees described how they used to work in Lebanon and send money home, but that their living costs and responsibilities increased when their families left Syria and joined them:

I used to come from work and go get some fresh air by the sea, but now, no… They think they made my life more difficult…they did, but they are my family and I am responsible. (male, aged 42).

Women also noticed a change in the behaviour of their husbands who struggled to fulfil their traditional duty as the main provider of their family’s material needs:

His life was different before…he used to take care of himself and get clothes but now he does not. He never gets anything new for himself now unless he receives second-hand clothes from people. The children are a priority.” (female, aged 44).

Women living in Shatila had lost sense of the things they previously enjoyed, including new clothes, wearing make-up, visiting friends and socialising and some were employed for the first time in their lives.

#### *“He used to be like a guest.”* changes in relationship dynamics

Shatila was the first place many participants had lived together as a couple for an extended period of time, as many men had been working in Lebanon prior to the war and only saw their families every few months. Their wives and children joined them in Lebanon later when they fled the war.

Different reactions to this new situation emerged during the interviews. Many participants felt that their relationships were under increased strain in Shatila, with some men abandoning their family responsibilities as they felt unable to cope with them. A 37 year old woman described how she first met her husband through this circumstantial reunification and was finding the situation difficult.

In other interviews however, relationships were described to have improved as husbands and wives felt a sense of solidarity as a result of their difficult living situation, and were able to support each other:

This is what happens when people are put in tough situations…they get to know each other a lot better. (male, aged 61).

Men such as P13 felt unable to meet the expectations of their families:

I feel like I am not fulfilling my responsibilities toward my family, so I cannot be angry at them…the shortcomings are from my side. [M]y wife once had a severe headache, but she refused to go to a doctor…she was willing to die so as not to ask me and have me say ‘*I don’t have the money.’* (male, aged 59).

These two women talked about how they had good relationships with their husbands since living together in Shatila:

Honestly, my husband is good with me. He loves me, he takes care of me and puts up with me. Even if I yell at him he has not reacted. (female, aged 44).

In Syria it was more difficult for me because my husband was in a different place, but here I feel more comfortable with him next to me and the children. (female, aged 40).

#### “*I feel that I’ve lost them”:* changes in children’s behaviour

Male and female participants had observed changes in their children since displacement, including increased violence, use of bad language and being more demanding and disrespectful. Many parents could not afford the school fees for their children, and some were sent to try and find work by their parents instead:

During the period that my husband had his operation, I did not know what to do so I had to make my children work selling tissues in the street, but they told me they did not know how to do it and they came back beaten every day. (female, aged 38).

At first they used to obey me, now they don’t…maybe because they are working now and generating money for our daily living. (male, aged 44).

Whilst some children took on additional responsibilities and left the home to work, in most cases, parents were over-protective of their children and did not allow them to go outside unaccompanied because of their concerns about insecurity.

This parent also noticed how their child began to withdraw from others and develop psychological symptoms:

My 6 year old son always wants to be alone, he doesn’t like to mix with people. When people come to visit us he goes and sits in a different room… He does not want to see anyone or mix with children his age. He always cries and shouts and he had urinary incontinence. (female, aged 38).

### Experiences of violence

#### “*I yell at them and hit them*”: parental violence against children

There were multiple examples of men and women reporting increased physical violence towards their children since displacement, which they linked to psychological suffering, stress and frustration:

Once I was approached by my son over having a fight with his friend… I got so angry that I hit him…I wanted to avoid this turning into a fight between grown-ups. (male, aged 59).

Now I’m much more strict with them than before. Sometimes I resort to hitting them when they misbehave or make noise. I need to rest at home. (male, aged 44).

One woman described how she punished her son for stealing from the vegetable market:

The people that my son was working for are people who make children steal. They steal vegetables from the market. I had to bring him back home. I tied him up with a chain and I hit him. (female, aged 37).

One participant and her husband both described being violent towards their son after moving to Shatila:

My husband always gets mad at the young boy…he yells at him and hits him. He comes home stressed from work and from the situation here. My husband was not like this before. I changed too. I used to get very stressed and hit them too…I did not used to be like this before. This is because of the pressure. I’m a different person now. I yell at them and hit them. (female, aged 44).

#### “I’m living the hardest life ever”: violence against women

There were several examples in our interviews in which women described how their husbands were physically violent towards them since displacement. One man also told us how he had beaten his wife.

Violence included shouting, verbal aggression, threats and physical violence.

He never hits me, but sometimes he says words that hurt more than hitting…we get very stressed. (female, aged 26).

My husband hit me in front of the children and he once told me that we have to leave for Syria, but I was like, ‘*fine, I’m not leaving’*. (female, aged 38).

One woman described an extremely violent relationship with her husband that she felt she could not escape from:

My husband once held three chairs and broke them on me. He hit me with a cane of whatever… Once he hit me with the hookah [pipe]. He does that because he knows my weakness…that I have nowhere to go and nobody to go to. (female, aged 19).

In addition to women experiencing violence from their husbands, participants also found the camp environment very violent and threatening and were afraid of going outside alone. One woman described how one man had thrown underwear at her face when she went outside, and was scared that it could happen again.

### Mental health

#### “*Psychologically, we are not well*”: anxiety, depression, fear and frustration

Participants were asked how they perceived the different experiences in Shatila to have affected their mental health. Many participants were extremely emotional during the interviews, and some cried when describing their exhaustion, anxiety, stress, fear, sadness and feelings of ‘*suffocation*’. Two men, including the one cited below, said their hair had ‘*turned white’* because of the stress they were under.

These 7 years are worth our entire lives. I did not have white hair in my beard, they grew in those 7 years. (male, aged 42).

Women in particular talked about how they neglected themselves and their physical appearance, with one 37 year old woman stating that she doesn’t “*take care of [her] beauty as a woman anymore*.” Her words were supported by those of another female interviewee:

I used to get dressed and always wore makeup…now I can’t do this; I’m not in the mood. Sometimes my children see old pictures of mine and start asking what changed and why I’m not like this now. (female, aged 25).

One participant described how her psychological distress affected her parenting skills:

I had psychological pressure and depression. I used to leave the house and go out…even my kids, I used to leave them. I neglected them and did not cook for them. I used to cry when I looked at them. I didn’t even brush their hair. I felt like my life was over, I hated my life, I stopped eating. (female, aged 44).

One 37 year old woman expressed symptoms suggestive of severe depressive disorder following the loss of her son in an accident in Shatila:

I did not think that the death of my eldest son was the major disaster…but what my other children are going through is. I don’t feel that I’m living anymore…life is meaningless for me now. It is like I’m searching for death and I can’t find it. Sometimes I feel that I want to leave everything and run away.

The development of psychotic features was reported by two participants – one talking about herself, and one about her daughter - after they had experienced extremely difficult life events during displacement. When men were asked about their mental health, they reported keeping their negative feelings ‘inside’.

#### “*I don’t have a future anymore”:* feelings of hopelessness and a lack of future perspective

When asked about their future in Shatila, all interviewees expressed feelings of hopelessness and feeling trapped and some expressed thoughts of suicidal ideation. They did not see a future for themselves or their children outside of the camp:

There’s no future here. I say it is the beginning of the end. No child will have a good future. (male, aged 42).

One 32 year old woman described how her and her husband felt at times that death was the only way out of Shatila and their current living conditions:

Sometimes I wish I could die to get rid of this life we are living. My husband is tired too. He often tells me that it’s better for him if he dies or if a car hits him.

Another interviewee described similar feelings:

Honestly I don’t feel that I’m living, life is meaningless for me now. It’s like I’m searching for death but can’t find it…it’s the same for my children whether I’m alive or dead. (female, aged 37).

For many participants, returning to Syria was preferable to staying in Shatila, but they expressed their concerns about having lost their homes and everything they owned during bombing and shelling, and in the case of men, feared conscription:

If I could go back, I would; yet I cannot because once I’m back I’ll be forced to serve in the army…then what would happen to my wife and children? (male, aged 31).

We are lost; it’s not safe in Syria yet and in Lebanon my husband’s not working…we feel we’re in the middle and we can’t move forwards or backwards. (female, aged 26).

## Discussion

Our qualitative study has described i) the conditions experienced by male and female Syrian refugees living in Shatila refugee camp for prolonged periods of time ii) gender and family dynamics, iii) expereinces of violence at home and in the community and iv) experiences of mental health issues within this population.

Seven years on from the start of the Syrian crisis in 2011, our findings about living conditions are similar to other earlier studies conducted with Syrian refugees in Lebanon, which also show that men and women suffer from harsh, cramped living conditions; unemployment; the daily struggle to find food; ongoing economic challenges and exorbitant living costs [22].

The repeated use of the word ‘*prison*’ and descriptions of feeling ‘*trapped*’ and ‘*suffocated*’ to describe Shatila were striking, and suggest living conditions that resemble institutional detention or imprisonment ([6, 7], Arsenejevic et al. 2018, [9]). Such feelings of being trapped were also expressed by participants in this study, and in other studies, to describe their position between a host country that does not welcome them and Syria, their country, to which they desire to return but cannot [30].

Harassment and lack of support from other people residing within Shatila were also commonly described by the participants in this study. This finding was also acknowledged in a recent study on Syrian refugees in Shatila camp in which host populations linked ‘hostility’ towards refugees to tension around jobs, access to health care and schools in an already severely impoverished camp [21].

Moreover, we found it interesting to note that contrary to other literature on refugee communities in contexts such as Southern Africa, where refugee populations often have strong, supportive bonds between them, there was little solidarity amongst Syrian refugees in Shatila. Similar experiences have been reported elsewhere amongst the Palestinian community following the refugee crisis and physical scattering of families in 1948 [10].

Participants did not have many social networks or support structures outside of their direct family, and were in competition with others in the camp for opportunities and resources, from employment to discarded vegetables in the market. In addition to a scarcity of resources, our interviewees also felt that there were enhanced degrees of mistrust amongst Syrians due to the polarising nature of the war. Moreover, people felt uncomfortable hosting visitors given their cramped living conditions and their extremely small apartments, as well as the lack of cafes or other gathering places inside the camp, which further reduced their opportunities to socialise. Many of the interviweees also described anxio-depressive symptoms which were linked to feelings of social isolation. This lack of support networks leads in turn to increased feelings of isolation and self-centeredness; a stark difference with the traditional social attitude and behavior of Syrians pre-war and the strong friendship and familial bonds men and women had in their home country.

There were very clear differences in the ways that men and women described their responsibilities and gender roles before and after displacement which have been highlighted in Lebanon and other contexts [8]. Some studies on Syrian refugees have noted this change in gender roles and likened it to ‘*more liberal gender roles of Lebanese society’* ([5], p. 29).

More than one interviewee described how women were taking on male and female roles at the same time, suggesting that displacement had challenged the gendered roles and responsibilities they had in Syria. We suggest that this change in gender roles puts men and women under increased pressure and can challenge family dynamics, which in turn may have increased tensions within families and the likelihood of violence [8, 13]. Men had traditionally been responsible for income generation and financial support in the household, as is common in Syrian culture, but the limited employment and housing options in Shatila left them frustrated at being unable to fulfill these responsibilities. Women were able to meet their childcare and household responsibilities more easily in Syria because of their access to support networks and resources. This led to feelings of insecurity and instability in Shatila. In addition to men and women taking on new roles and responsibilities as a consequence of prolonged displacement, children also found themselves with additional tasks to carry out, such as contributing economically to the household, and were no longer able to attend school.

Whilst these economic pressures and insecure living environment increased tensions within almost all families in our study, what was interesting to note was that some interviewees reported how their environment had in fact brought husbands and wives closer together. This has also been documented amongst refugees in Afghanistan and Somalia [3, 17]. In our study, the increased bond was largely due to married couples living together for the first time, and supporting each other through an incredibly difficult and stressful situation without larger community networks.

Whilst our interviewees did not discuss as many cases of violence against women as we expected based on literature about gender-based violence in conflict and amongst refugee populations [24, 31], many described physical violence towards children as a result of their cramped living conditions, frustrations and sense of imprisonment within the camp, resembling findings in some other studies [2]. We must also consider, as discussed in the limitations below, that some men and women did not feel comfortable discussing gender-based violence during the interviews, thus may have been under-reporting it.

Violence was, however, clearly experienced through the continuous harassment, discrimination, bullying, humiliation and witnessing of violence in Shatila’s streets as well as some participants reliving the bombing, shelling and abuse which occurred in Syria. This fear led to parents becoming over-protective of their children, and in more cases than we expected, they resorted to violence to discipline, punish and control their children; a similar finding to a vulnerability study among Syrian refugees in Lebanon where 78% of children were found to be subject to violent discipline [27]. The over-protection of children was also noted in a similar paper from Lebanon as well as in other contexts where people have been displaced [5, 22].

In relation to mental health, male and female participants described depression and anxiety manifesting in feelings of immense sadness, frustration, hopelessness, social withdrawal, self-neglect and a loss of sense of self and self-worth. Such manifestations of psychological distress among Syrian and other refugees have been reported elsewhere as the result of ongoing violence, displacement, changes in gender and family roles, and harsh living conditions [13]. The fact that most Syrian refugees in Shatila came from green spacious rural areas yet ended up feeling locked in the camp has also deeply affected their mental wellbeing. Several participants expressed the wish to end their life so as to escape their current situation, and imagined death as the only alternative to living in Shatila. Similar expressions of suicidal thoughts and self-harm have been reported elsewhere in Lebanon [23] and amongst other refugee populations [1, 9].

The findings of this study will be important to consider in current or future mental health programs targeting Syrian refugees in Shatila camp or other similar settings. We have several recommendations for the provision of mental health services in Shatila on the basis of our research findings. It is essential to adopt an approach towards the provision of mental health care which is tailored towards long-term displacement and systematic exposure to traumatic events. Any services should also consider and treat the family as a unit, rather than only as individuals, in order to rebuild fragmented relationships and build on the strong bonds which some couples have developed.

We also see the need for a community-based approach that includes psycho-social activities, rather than being limited to clinic-based therapy, particularly considering the security concerns expressed by interviewees. There is the need to address parental violence towards children as well as violence towards women from their husbands, through developing or adapting scales that systematically screen for parental and gender-based violence. Organising psycho-social and awareness activities for families and children in and out of school could help address the problem of violence towards children. We could also consider enhancing outreach activities for mental health services with men, including implementing services outside of working hours in order to reach those who are seeking employment or working long shifts. In addition, further research to explore men’s perceived barriers and facilitators to accessing mental health services in this context will be of definite value for more tailored mental health interventions.

There are several strengths and limitations to this study. Our study gave us unique access to the stories of female mental health patients and men in the wider Shatila community, and to our knowledge, is the first study looking at gender, violence and mental health in Syrian refugees in this context. Whilst we are able to include the voices of men and women, we were unable to include male mental health patients as there are so few male beneficiaries of MSF’s mental health services. We must also be aware that as female interviewees were beneficiaries of MSF’s services, and men were aware of MSF in the community, they may have been reluctant to criticise MSF’s services or say that they required more health support, despite being reassured during the consent procedure that their participation would not affect their access to care.

We should also consider that as women were recruited from the mental health clinic, we may see more mental health problems amongst this group of women than amongst women in the wider community. However, those who attend mental health services are arguably not the most vulnerable, as they have already accessed support.

Another limitation was that the PI is female, which may have meant that some men were reluctant to open up during interviews for fear of being seen as ‘weak’. Men were offered the choice of having a male present during interviews if they wished, but only one accepted this offer. We are also aware of the limitation with regard to the potential under-reporting of violence, from both genders but especially men.

## Conclusions

This paper has presented participants’ perceptions of their traditional gender roles, family dynamics and experience of violence after lengthy displacement in harsh living conditions in Shatila camp, as well as descriptions of their mental health concerns. Most participants perceived Shatila as unsafe and insecure; and as such preferred to imprison themselves and their families within their small, cramped one-room houses, which in turn led to enhanced feelings of isolation. Parental violence was the most common type of domestic violence reported, linked to frustration and feeling powerless as a result of their current situation and living conditions. The symptoms of mental illness described by the Syrian refugees in our study is worrying, and we believe that the findings of our study will be important to consider in current or future mental health programs targeting Syrian refugees in Shatila and other similar contexts.

### Supplementary information

**Additional file 1:** In-depth interview guide for female participants. (DOCX 22 kb)

**Additional file 2:** In-depth interview guide for male participants. (DOCX 22 kb)

## Data Availability

The datasets used and/or analysed during the current study are available from the corresponding author on reasonable request.

## Appendix

**Table 1:** Demographic profile of study participants

Participant number	Gender	Age	Duration of displacement (years)	Number of children
1	Female	37	5	4
2	Male	42	4	6
3	Female	44	5	6
4	Female	37	5	4
5	Female	25	2	5
6	Female	40	6	6
7	Female	26	4	2
8	Female	37	6	5
9	Female	38	4	4
10	Female	37	4	3
11	Female	32	3	3
12	Male	61	7	7
13	Male	59	5	3
14	Male	35	3	4
15	Male	35	2	3
16	Male	25	3	0
17	Male	31	3	2
18	Male	44	4	7
19	Male	23	4	1
20	Female	19	2	1
21	Male	52	4	6
22	Female	31	4	6
23	Female	33	3	6
24	Male	38	7	1
25	Male	36	6	3
26	Male	21	7	1
27	Male	32	4	3
28	Male	37	2	2

## Abbreviations

ICRC: International Committee of the Red Cross
IDI: In-depth interview
MSF: Médecins Sans Frontières
PHC: Primary Health Care
PI: Principal investigator
WHC: Women’s Health Center
