# Supplementary material for: “With every passing day I feel like a candle, melting little by little.” experiences of long-term displacement amongst Syrian refugees in Shatila, Lebanon
Source: Confl Health. 2019 Oct 10;13:45. doi: 10.1186/s13031-019-0228-7 (PMC6785930; doi:10.1186/s13031-019-0228-7)
Supplement: Supplementary file 1 — Additional file 1: In-depth interview guide for female participants. (DOCX 22 kb) [file 13031_2019_228_MOESM1_ESM.docx]

# Additional file 1: In-depth interview guide for female participants

**Interview guide for female participants in Shatila**

Thank you for agreeing to talk to me today. As discussed in the informed consent, everything you tell me will remain confidential, and there are no right or wrong answers to my questions. You are free to stop the interview at any time and do not have to answer any questions that make you feel uncomfortable or upset.

**Q1:** Can you tell me a little bit about yourself?

**Prompts:** Marital status, living arrangements, family background and number of children, length of time in Lebanon, places lived in Lebanon

**Q2:** Can you tell me about your experience living in Shatila? What are the things you find most difficult about life here?

**Prompts:** Overcrowding, sanitation, salt-water, trash, electricity, employment, health-care, education

**Q3:** How is your life at present different from your life in Syria?

**Prompts:** Employment, living conditions, education, family life and relationships

**Q4:** Can you tell me what life is like for women in Syria? How is your current life as a woman in Shatila different from the one you had in Syria?

**Prompts:** change in roles, change in relationship with husband, different household composition, change in responsibilities

**Q5:** Do you think life is different for your husband, compared to life in Syria, and how? How do you feel about these changes, if any?

**Prompts:** Has anything changed in your relationship with him? When did you start noticing these changes? Has anything been getting better/worse over time?

**Q6:** Some men in this community talk about facing difficulties with their situation since displacement. How does your husband react to these changes?

**Prompt:** Frustration, anger, violence towards interviewee or children, anger, changes in mood

**Q7:** Have you noticed any changes in the behaviour and mood of your children since you came to Shatila? Can you give examples?

**Prompt:** How are you dealing with the changes? Have you or your husband been more aggressive toward them? Please give examples.

**Q8:** How do the things you’ve described today affect you? Can you explain to me why you sought mental health services?

**Q9:** Do you have any specific coping mechanisms you use to help you in your daily life? Do you have any ways of coping which you think are good or bad, and can you describe them?

**Q10:** Do you have any thoughts about your future? How do you see your situation evolving?

I thank you for taking the time to answer these questions and assist me with my research. Do you have any questions you would like to ask me?
