# Supplementary material for: “With every passing day I feel like a candle, melting little by little.” experiences of long-term displacement amongst Syrian refugees in Shatila, Lebanon
Source: Confl Health. 2019 Oct 10;13:45. doi: 10.1186/s13031-019-0228-7 (PMC6785930; doi:10.1186/s13031-019-0228-7)
Supplement: Supplementary file 2 — Additional file 2: In-depth interview guide for male participants. (DOCX 22 kb) [file 13031_2019_228_MOESM2_ESM.docx]

# Additional file 2: In-depth interview guide for male participants

**In-depth interview guide for male participants in Shatila**

Thank you for agreeing to talk to me today.

As discussed in the informed consent, everything you tell me, as a researcher, will remain confidential, and there are no right or wrong answers to my questions. You are free to leave the interview at any time and do not have to answer any questions that make you feel uncomfortable or upset.

**Q1**: Can you tell me a little bit about yourself?

**Prompts**: Marital status, living arrangements, family background and number of children, length of time in Lebanon, places lived in Lebanon.

**Q2:** Can you tell me about your experiences living in Shatila? What are the things you find most difficult about life here?

**Prompts:** Overcrowding, sanitation, salt-water, trash, electricity, employment and money, health-care, education

**Q3:** How is life at present, in Shatila, different from your life in Syria?

**Prompts:** Employment, living conditions, education, family life and relationships, social life

**Q4:** Can you tell me what life is like for men in Syria? How is life as a man in Shatila different from life in Syria?

**Prompts:** change in roles, change in relationship with wife, different household composition, change in responsibilities

**Q5:** Do you think life is different for women in Shatila compared to life in Syria, and how? How do you think women, such as your wife or sister, feel about these changes?

**Prompts:** Has anything changed in your relationship with your wife? When did you start noticing these changes? Has anything been getting better/worse over time?

**Q6:** Some men in this community talk about finding their current situation challenging. Is this something that you have experienced? How do you cope with these challenges?

**Prompt:** Aggressive behaviour, aggression at home, frustration

**Q7:** Has your family life changed since you came to Lebanon, and if so, how?

**Prompts:** Changes in relationship with wife, change in behaviour of children, changes for better or worse over time

**Q8:** How do the things you’ve described today affect you? How do men such as yourself cope with all the challenges you described above?

**Q9:** Do you have any thoughts about your future in Shatila? How do you see the situation here evolving?

I thank you for taking the time to answer these questions and assist me with my research. Do you have any questions you would like to ask me?
